# Nanoparticle-Based Lateral Flow Biosensor Integrated With Loop-Mediated Isothermal Amplification for Rapid and Visual Identification of *Chlamydia trachomatis* for Point-of-Care Use

**DOI:** 10.3389/fmicb.2022.914620

**Published:** 2022-07-12

**Authors:** Xu Chen, Qingxue Zhou, Yan Tan, Ronghua Wang, Xueli Wu, Jiangli Liu, Rui Liu, Shuoshi Wang, Shilei Dong

**Affiliations:** ^1^The Second Clinical College, Guizhou University of Traditional Chinese Medicine, Guiyang, China; ^2^Clinical Medical Laboratory of the Second Affiliated Hospital, Guizhou University of Traditional Chinese Medicine, Guiyang, China; ^3^Clinical Laboratory, Hangzhou Women's Hospital, Hangzhou, China; ^4^Guizhou Provincial Center for Clinical Laboratory, Guiyang, China; ^5^Department of Clinical Laboratory, Longli People's Hospital, Qiannan Buyi and Miao Autonomous Prefecture, China; ^6^Department of Clinical Laboratory, Zhejiang Hospital, Hangzhou, China

**Keywords:** *Chlamydia trachomatis*, loop-mediated isothermal amplification, gold nanoparticle-based lateral flow biosensor, limit of detection, point-of-care testing

## Abstract

Chlamydial infection, caused by *Chlamydia trachomatis*, is the most common bacterial sexually transmitted infection and remains a major public health problem worldwide, particularly in underdeveloped regions. Developing a rapid and sensitive point-of-care (POC) testing for accurate screening of *C. trachomatis* infection is critical for earlier treatment to prevent transmission. In this study, a novel diagnostic assay, loop-mediated isothermal amplification integrated with gold nanoparticle-based lateral flow biosensor (LAMP-LFB), was devised and applied for diagnosis of *C. trachomatis* in clinical samples. A set of LAMP primers based on the *ompA* gene from 14 *C. trachomatis* serological variants (serovar A-K, L1, L2, L3) was successfully designed and used for the development of *C. trachomatis*-LAMP-LFB assay. The optimal reaction system can be performed at a constant temperature of 67°C for 35 min. The total assay process, including genomic DNA extraction (~15 min), LAMP reaction (35 min), and LFB readout (~2 min), could be finished within 60 min. The *C. trachomatis*-LAMP-LFB could detect down to 50 copies/ml, and the specificity was 100%, no cross-reactions with other pathogens were observed. Hence, our *C. trachomatis*-LAMP-LFB was a rapid, reliable, sensitive, cost-effective, and easy-to-operate assay, which could offer an attractive POC testing tool for chlamydial infection screening, especially in resource starvation settings.

## Introduction

*Chlamydia trachomatis* is a Gram-negative, obligate intracellular pathogen that causes the most common bacterial sexually transmitted infections (STIs) (Woodhall et al., 2018; Murray and McKay, 2021), with an estimated ~130 million new cases worldwide each year, thereby remaining a major global health challenge, particularly in underdeveloped regions (World Health Organization, 2016). *C. trachomatis* infection in women can cause pelvic inflammatory disease, including salpingitis, tubo-ovarian abscesses, endometritis, pelvic peritonitis, and cervical carcinoma (Cooksey et al., 2010; Di Pietro et al., 2019). Also, chlamydial infections during pregnancy can lead to adverse outcomes, such as premature rupture of membranes, stillbirth, miscarriages, and preterm labor with low birth weight (Adachi et al., 2016). Maternal transmission to neonate during birth may also cause infant pneumonia, otitis media, and inclusion conjunctivitis (Wise et al., 2015). Chlamydial infection in men can lead to orchitis, epididymitis, and urethritis (Dukers-Muijrers et al., 2016; Rowley et al., 2019). Furthermore, *C. trachomatis* infection is related to an increased risk of human immunodeficiency virus (HIV) infection and transmission (Khosropour et al., 2021). In addition, ocular infection with *C. trachomatis* can bring about trachoma, which is the leading cause of blindness worldwide (Pourabbas et al., 2018; Khosropour et al., 2021). Owing to the majority of patients being asymptomatic, making early diagnosis is difficult (Park et al., 2021). Developing a rapid and sensitive point-of-care (POC) testing for accurate screening of *C. trachomatis* infection is critical for earlier treatment to prevent transmission.

Traditional laboratory-based diagnosis of urogenital *C. trachomatis* infection was based on cell cultivation (Meyer, 2016). Isolating pathogens from cell cultures was considered as the initial gold standard for diagnosis of *C. trachomatis* (Meyer, 2016; Kelly et al., 2017). However, the sensitivity was low (ranging from 60 to 80%) and requires stringent conditions in specimen collection, transport, and cultivation (Peng et al., 2020). In addition, cell culture is labor-intensive and time-consuming (48–72 h). Hence, it is rarely performed in routine clinical diagnostic application. Nucleic acid amplification technologies (NAATs), including polymerase chain reaction (PCR) and ligase chain reaction, have been considered as a major breakthrough and have been regarded as the new gold standard for the detection of *C. trachomatis* infection for high sensitivity, specificity, and automation (Gaydos et al., 2013; Safarkar et al., 2017). Nevertheless, NAATs are often unaffordable and inaccessible in resource-limited regions due to requiring skilled technicians and expensive analytical instruments. Therefore, devising a cheap, rapid, simple, and accurate assay is critical to making an early diagnosis and follow-up antibiotic use during the initial stage of *C. trachomatis* infection.

Loop-mediated isothermal amplification (LAMP), an innovative nucleic acid isothermal amplification technique, was considered as an attractive alternative to traditional NAATs, which has potential applications as a POC testing for its simplicity, rapidity, and easy-to-operate (Notomi et al., 2000; Sahoo et al., 2016; Shirato, 2019). More importantly, it has been widely applied to diagnosis of various pathogens, such as SARS-CoV-2, *Neisseria gonorrhoeae*, and HIV (Rudolph et al., 2015; Chen et al., 2021a,b). LAMP amplicons can be analyzed with electrophoresis, turbidity, fluorescent dye, and colorimetric indicators (Li et al., 2017; Shirato, 2019). Nevertheless, all of these techniques require special facilities and reagents. To conveniently detect the LAMP products, gold nanoparticle-based lateral flow biosensor (LFB) is used as a paper-based and POC testing platform, which is currently of enormous potential for clinical applications due to its rapidness, visualization, simplicity, cost-saving, and portability of detection of molecular markers (Anfossi et al., 2019; Ye et al., 2020).

**Figure 1:**
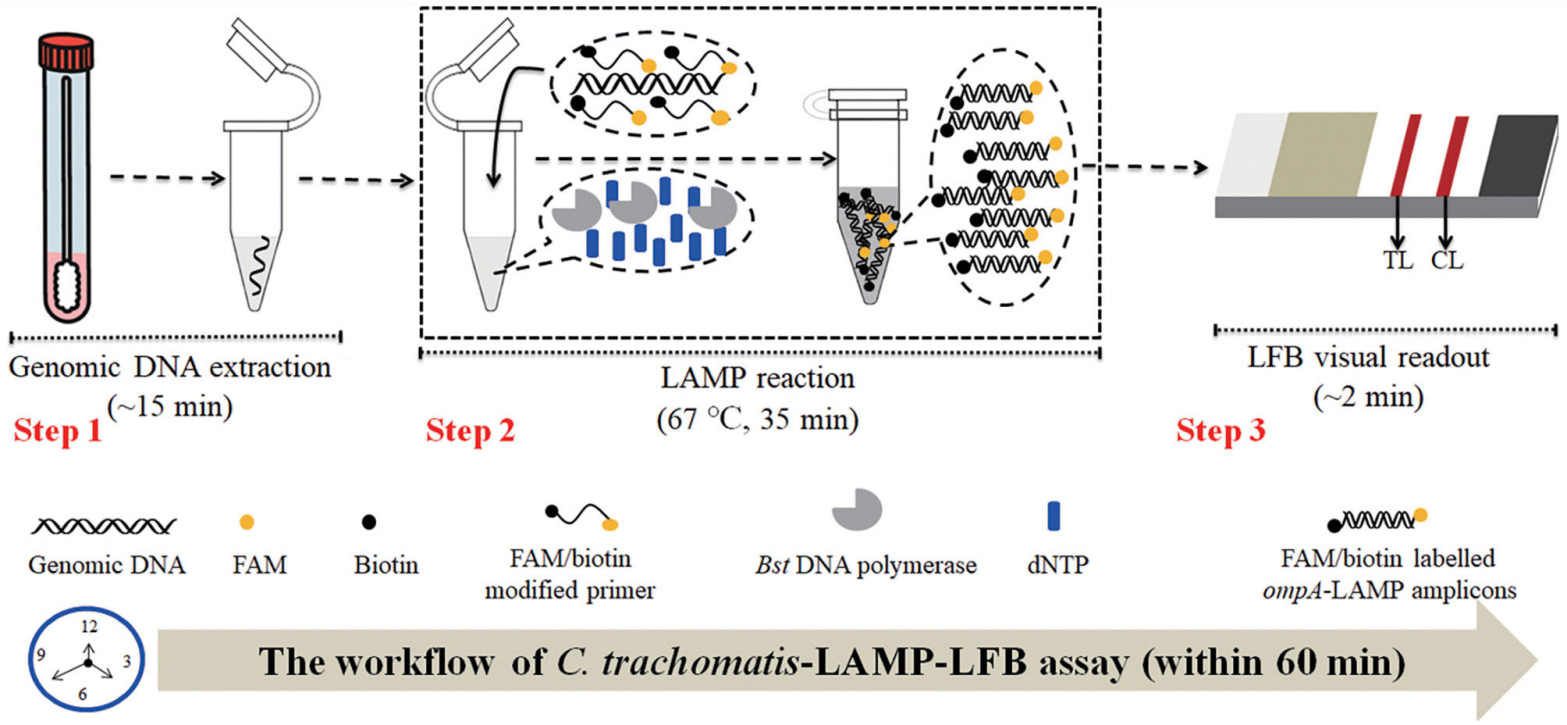
*C. trachomatis*-LAMP-LFB workflow. *C. trachomatis*-LAMP-LFB assay contains three steps: genomic DNA preparation (step 1), LAMP reaction (step 2), and LFB visually readout (step 3). The whole diagnosis procedure can be completed within 60 min.

In this study, LAMP integrated with gold nanoparticle-based LFB (LAMP-LFB) was first developed for visual, rapid, specific, sensitive, portable, identification of *C. trachomatis* by targeting the *ompA* gene (Molano et al., 2018; Somboonna et al., 2018). The principle of *C. trachomatis*-LAMP-LFB is illustrated in Figure 1, and its feasibility was confirmed with clinical samples. The assay was completed with a high accuracy within 60 min. Therefore, the LAMP-LFB method can be regarded as a valuable POC assay for the detection of *C. trachomatis* infection, particularly in resource-scarce regions.

## Materials and Methods

### Reagents

**Figure 2:**
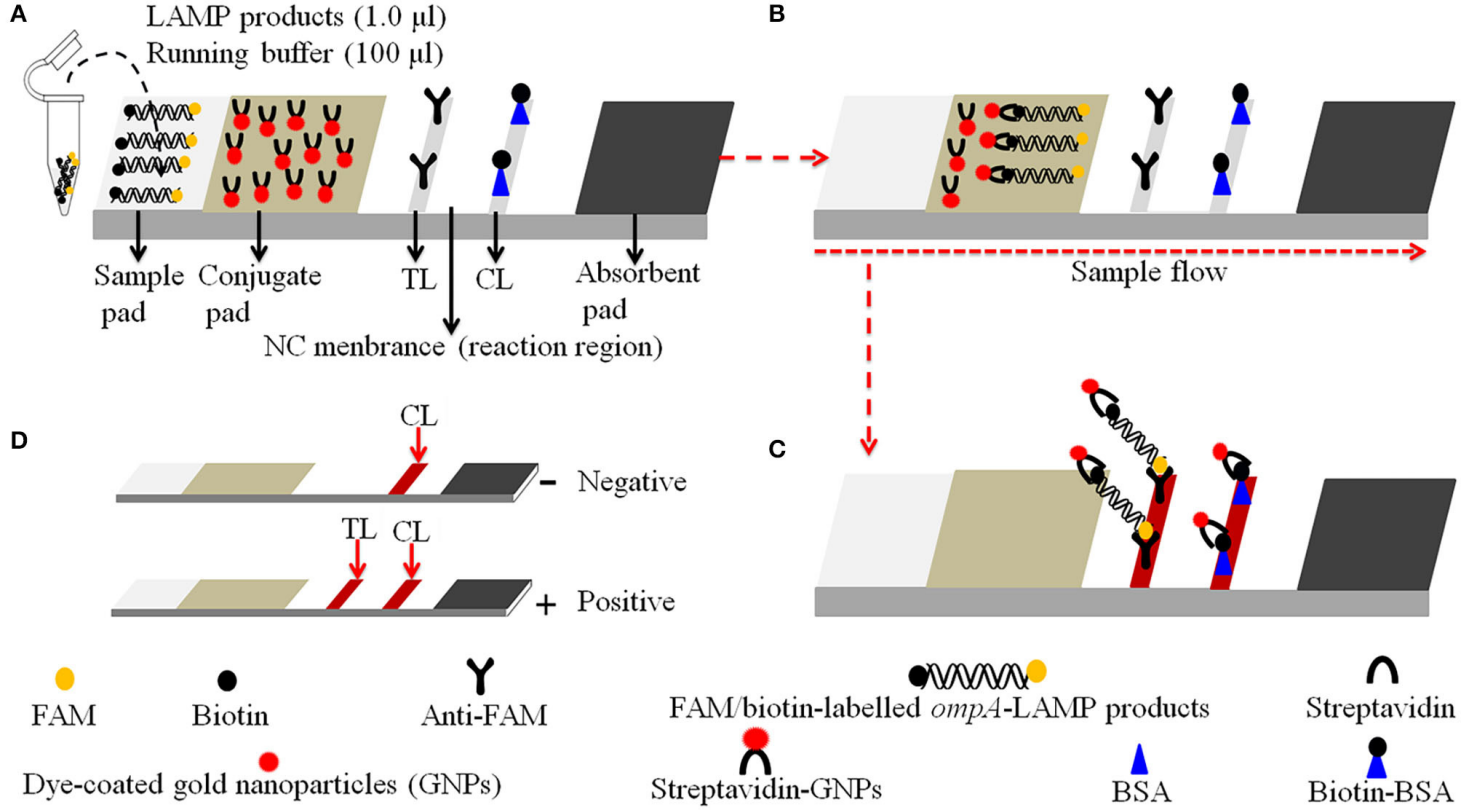
Schematic diagram of the principle of LFB for visually readout *C. trachomatis*-LAMP products. **(A)** *C. trachomatis*-LAMP products and (1.0 μl) and running buffer (100 μl) were simultaneously added to the sample pad. **(B)** The running buffer and *C. trachomatis*-LAMP products move forward to conjugate pad and reaction region due to capillary action. **(C)** For positive results, the FAM/biotin-labeled *ompA*-LAMP products are arrested by anti-FAM at TL strip, and the streptavidin-DPNs are arrested through biotin-BSA at CL strip. For negative results, only the streptavidin-DPNs flow to reaction region and arrested by biotin-BSA at CL strip. **(D)** Interpretation of the *C. trachomatis*-LAMP-LFB assay results: negative—only the CL appears on the LFB; positive—CL and TL appear on biosensor.

The LAMP kits and visual detection reagents (Malachite Green, MG) were obtained from HuiDeXin Bio-technology (Tianjin, China). Nucleic acid-releasing agents were purchased from Sansure Biotech Inc. (Changsha, China). Dye streptavidin-coated gold nanoparticles (size: 35 ± 5 nm; crimson red) were obtained from Bangs Laboratories, Inc. (Indiana, USA). Rabbit anti-fluorescein antibody (anti-FAM) and biotinylated bovine serum albumin (biotin-BSA) were purchased from Abcam Co., Ltd. (Shanghai, China). Gold nanoparticle-based biosensor materials, including sample pad, conjugate pad, nitrocellulose (NC) membrane, and absorbent pad were manufactured and assembled by HuiDeXing Biotech. Co., Ltd. (Tianjing, China) according to the design instructions of this study (Figure 2).

### Clinical Specimens and Target Genes Preparation

A total of 87 clinical genital secretion samples were collected from suspected *C. trachomatis*-infected patients at the Hangzhou Women's Hospital between July 2021 and December 2021. The genomic DNA was obtained using nucleic acid-releasing agents (Sansure Biotech, Changsha, China) in accordance with the instructions of the manufacturer. Briefly, the collected genital secretion samples were centrifuged at 12,000 rpm for 5 min. The pellet was suspended in 50 μl nucleic acid-releasing agents and incubated at room temperature (25°C) for 10 min, and the supernatant was used as templates for assay. The concentration of nucleic acid was measured using Nano-Drop ND-2000 (Beijing, China) at A260/280.

The full-length DNA sequences of *ompA* gene for *C. trachomatis* serological variants A-K, L1, L2, L3 (serovar A: GenBank Accession No. JX548318.1; serovar B: GenBank Accession No. JX559518.1; serovar C: GenBank Accession No. JX559519.1; serovar D: GenBank Accession No. KP164991.1; serovar E: GenBank Accession No. JX559522.1; serovar F: GenBank Accession No. JX564244.1; serovar G: GenBank Accession No. JX564245.1; serovar H: GenBank Accession No. JX564246.1; serovar I: GenBank Accession No. JX564247.1; serovar J: GenBank Accession No. JX648604.1; serovar K: GenBank Accession No. JX564248.1; serovar L1: GenBank Accession No. JX569832.1; serovar L2: GenBank Accession No. KP120855.1; serovar L3: GenBank Accession No. JX569834.1) were synthesized and cloned in pUC57 vector through Tsingke Biotech (Beijing, China), respectively. The initial concentration of each plasmid was 1 × 10^8^ copies/ml, and the *C. trachomatis* serovar A-plasmid was used as a positive control.

### Gold Nanoparticle-Based Biosensor Preparation

The schematic design of gold nanoparticle-based LFB is shown in Figure 2. Briefly, the LFB consisted of four sections, namely, sample pad, conjugate pad, NC membrane with immobilized anti-FAM and biotin-BSA, and an absorbent pad, all of them were assembled on a plastic adhesive backing card (HuiDeXin Bio-technology, Tianjin, China). Dye streptavidin-coated gold nanoparticles (GNPs) were deposited on the conjugate pad, the size of GNPs was 35 ± 5 nm, and the concentration was 10 mg/ml. Rabbit anti-FAM antibody (0.2 mg/ml; Abcam) and biotin-BSA (4 mg/ml; Abcam) were fixed onto the NC membrane as test line (TL) and control line (CL), respectively. Each line was separated by 5 mm. Hence, the LFB can detect two targets, including *C. trachomatis* LAMP amplicons (TL) and a chromatography control (CL).

### *Chlamydia trachomatis*-LAMP Primers Design and Screening

The *ompA* genes from 14 *C. trachomatis* serological variants (serovar A, B, C, D, E, F, G, H, I, J, K, L1, L2, L3) were aligned with MEGA-X (https://www.megasoftware.net/), and then the conserved sequences were selected for designing of *C. trachomatis*-LAMP primers. A total of three sets of LAMP primers were designed by Primer Explorer V5 (http://primerexplorer.jp/e/) according to the LAMP reaction mechanisms. Each set of LAMP primers includes outer primers F3 and B3, loop primers LF and LB, and inner primers FIP and BIP. The screening of primers was performed with standard *C. trachomatis*-LAMP reaction system, and LAMP amplicons were monitored using real-time turbidity (LA-500). The first set of LAMP primers showed better outcome in amplification efficiency (data not shown), which was used for the establishment of the *C. trachomatis*-LAMP assay. The schematic design of *C. trachomatis*-LAMP primers is demonstrated in Supplementary Figure 1, and the sequences and modifications are shown in Table 1. All LAMP primers were synthesized and purified by Tsingke Biotech (Beijing, China) at high-performance liquid chromatography purification grade.

**Table 1:** *C. trachomatis*-LAMP primers used in this study.

**Primer name**	**Sequence and modifications**	**Length**	**Gene**
F3	5′- GT(A/T)TTTGCCGCTTTGAGTTCTG-3′	22 nt	*ompA*
B3	5′- AAAC(A/G)CGGTCGAAAACAAAGTC-3′	22 nt	
FIP*	5′-FAM- ATTCCGTCGATCATAAGGCTTGGTCTTCCTCCTTGCAAGCTCTG-3′	44 mer	
BIP	5′-TGGGAAGGTTT(C/T)GG(C/T)GGAGAT(A/T)CC(A/G)TAGTAACC(A/C)A(C/T)(A/G)CGCATG-3′	43 mer	
LF*	5′-Biotin-CAGCAGGATTCCCCACA-3′	17 nt	
LB	5′-ATCCTTGC(A/G)CCACTTGGTG-3′	19 nt	

FIP*, 5′-labeled with FAM when applied for LAMP-LFB detection; LF*, 5′-labeled with biotin when applied for LAMP-LFB detection; FAM, 6-carboxy-fluorescein; nt, nucleotide; mer, monomeric unit.

### *Chlamydia trachomatis*-LAMP Reaction and Detection

The standard LAMP reaction was performed with an isothermal amplification kit (HuiDeXing Biotech. Co., Ltd. Tianjing, China). Briefly, a 25 μl reaction mixture contained 12.5 μl 2 × reaction buffer [16 mM MgSO_4_, 2 M betaine, 40 mM Tris–HCl (pH 8.8), 40 mM KCl, 20 mM (NH_4_)_2_SO_4_, and 0.2% Tween-20]; 1 μl of standard plasmid template (5 μl of clinical sample template); 0.4 μM each outer primer, F3 and B3; 0.8 μM each loop primer, LF^*^ and LB; 1.6 μM each inner primer, FIP^*^ and BIP; 8 U of *Bst* 2.0 DNA polymerase; and double-distilled water (DW) was added to 25 μl. The reaction was carried out in a heat block at constant temperature (optimization outlined later).

Real-time turbidity (LA-500), visual detection reagents (Malachite Green, MG), and LFB were applied to analyze the LAMP products and confirm the optimal *C. trachomatis*-LAMP-LFB reaction system. For the real-time turbidity detection, turbidity > 0.1 was considered as a positive result. For visual MG reagent analysis, the reaction mixtures changed to light green, indicating a positive outcome, and remained colorless, suggesting a negative result. For the LFB method, CL and TL were observed simultaneously, indicating a positive outcome, and only the CL appeared, suggesting a negative result.

### *Chlamydia trachomatis*-LAMP Reaction Temperature Optimization

Temperature is important for LAMP reaction. An optimal amplification temperature of *C. trachomatis*-LAMP ranging from 63 to 70°C (with 1°C intervals) was evaluated with *ompA*-plasmids (5.0 × 10^3^ copies/ml). The LAMP products were monitored with real-time turbidity (LA-500). The examinations were conducted independently in triplicate.

### Sensitivity of the *C. trachomatis*-LAMP-LFB Assay

The initial concentrations of *ompA* standard plasmids were 1 × 10^8^ copies/ml. Then, 10-fold serial diluted from 5.0 × 10^4^ to 5.0 × 10^−1^ copies/ml were used to evaluate the limit of detection (LoD) of the *C. trachomatis*-LAMP-LFB. The reactions were carried out under optimal reaction temperature, and the outcomes were analyzed simultaneously with MG and LFB. The LoD of *C. trachomatis*-LAMP was verified as the lowest dilution for which all three replicates were positive. Next, the concentration of *ompA*-plasmids at the LoD level was applied for identifying the optimal reaction times (15, 25, 35, and 45 min) of the *C. trachomatis*-LAMP assay.

### Specificity of the *C. trachomatis*-LAMP-LFB Assay

The analytical specificity of the *C. trachomatis*-LAMP-LFB was estimated by comparing *C. trachomatis* DNA templates (serovar A-K, L1, L2, L3) with the nucleic acid (at least 1.0 × 10^4^/test) extracted from various pathogens (Table 2), and DW was used as a blank control. The results were analyzed with LFB, and each test was performed in triplicate.

**Table 2:** Pathogens used in this study.

**No**.	**Pathogen**	**Source of pathogens^a^**	**No. of strains**	***C. trachomatis*-LAMP-LFB result^b^**
1	*C. trachomatis* serovar A *ompA*-plasmids	Constructed by Tsingke Biotech (Beijing, China)	1	P
2	*C. trachomatis* serovar B *ompA*-plasmids	Constructed by Tsingke Biotech (Beijing, China)	1	P
3	*C. trachomatis* serovar C *ompA*-plasmids	Constructed by Tsingke Biotech (Beijing, China)	1	P
4	*C. trachomatis* serovar D *ompA*-plasmids	Constructed by Tsingke Biotech (Beijing, China)	1	P
5	*C. trachomatis* serovar E *ompA*-plasmids	Constructed by Tsingke Biotech (Beijing, China)	1	P
6	*C. trachomatis* serovar F *ompA*-plasmids	Constructed by Tsingke Biotech (Beijing, China)	1	P
7	*C. trachomatis* serovar G *ompA*-plasmids	Constructed by Tsingke Biotech (Beijing, China)	1	P
8	*C. trachomatis* serovar H *ompA*-plasmids	Constructed by Tsingke Biotech (Beijing, China)	1	P
9	*C. trachomatis* serovar I *ompA*-plasmids	Constructed by Tsingke Biotech (Beijing, China)	1	P
10	*C. trachomatis* serovar J *ompA*-plasmids	Constructed by Tsingke Biotech (Beijing, China)	1	P
11	*C. trachomatis* serovar K *ompA*-plasmids	Constructed by Tsingke Biotech (Beijing, China)	1	P
12	*C. trachomatis* serovar L1 *ompA*-plasmids	Constructed by Tsingke Biotech (Beijing, China)	1	P
13	*C. trachomatis* serovar L2 *ompA*-plasmids	Constructed by Tsingke Biotech (Beijing, China)	1	P
14	*C. trachomatis* serovar L3 *ompA*-plasmids	Constructed by Tsingke Biotech (Beijing, China)	1	P
15	*C. trachomatis* (clinical samples)	Hangzhou Women's Hospital	5	P
16	*Neisseria gonorrhoeae*	Hangzhou Women's Hospital	1	N
17	*Ureaplasma urealyticum*	Hangzhou Women's Hospital	1	N
18	*Mycobacterium tuberculosis* (nucleic acid samples)	GZCDC	1	N
19	*Escherichia coli*	2nd GZUTCM	1	N
20	*Haemophilus influenza*	ATCC49247	1	N
21	*Cryptococcus neoformans*	ATCC13690	1	N
22	*Streptococcus pyogenes*	2nd GZUTCM	1	N
23	*Pseudomonas aeruginosa*	2nd GZUTCM	1	N
24	*Staphylococcus aureus*	2nd GZUTCM	1	N
25	*Candida glabrata*	2nd GZUTCM	1	N
26	*Bordetella pertussis*	GZCCL	1	N
27	*Hemophililus parainfluenza*	GZCCL	1	N
28	*Klebsiella pneumoniae*	GZCCL	1	N
29	*Mycoplasma pneumoniae*	Hangzhou Women's Hospital	1	N
30	*Shigella flexneri*	Hangzhou Women's Hospital	1	N
31	*Listeria monocytogenes*	GZCCL	1	N
32	Human enterovirus EV71	GZCCL	1	N
33	Coxsackie virus CAV16	GZCCL	1	N
34	Human rhinovirus	GZCCL	1	N
35	Human papilloma virus	GZCCL	1	N

a *2nd GZUTCM, the Second Affiliated Hospital, Guizhou University of Traditional Chinese Medicine; ATCC, American Type Culture Collection; GZCCL, Guizhou Provincial Center for Clinical Laboratory; GZCDC, Guizhou Provincial Center for Disease Control and Prevention*.

b *P, positive; N, negative*.

### Validating the Feasibility of *C. trachomatis*-LAMP-LFB Using Clinical Samples

For confirming assay feasibility, the optimized diagnostic system was verified with clinical specimens. A total of 87 suspected *C. trachomatis*-infected genital secretion samples were collected from Hangzhou Women's Hospital (Hangzhou, China). Specimens were simultaneously detected using real-time quantitative PCR (qPCR) and LAMP-LFB assays. qPCR diagnosis was performed with a commercial *C. trachomatis* nucleic acid assay kit (DaAn Gene Co., Ltd. China), and the amplification and detection were performed using Applied Biosystems™ 7500 Real-Time PCR System (Life Technologies, Singapore). The concentration of more than 500 copies/ml of *C. trachomatis* is regarded as a positive outcome according to the instructions of the manufacturer. The *C. trachomatis*-LAMP-LFB process is described above. The whole process was carried out at biosafety level 2 according to the *WHO Laboratory Biosafety Manual*, 3rd edition.

## Results

### Schematic Mechanism of the *C. trachomatis*-LAMP-LFB Assay

The *C. trachomatis*-LAMP-LFB schematic mechanism and workflow are shown in Figure 1. In brief, the *C. trachomatis* genomic DNA was released by nucleic acid releasing agents (**Step 1**), and then was pre-amplified through LAMP at a constant temperature of 67°C for 35 min, two core primers, including FIP and LF, were labeled at the 5′-end with FAM and biotin, respectively. As a result, plenty of detectable double-labeled products are formed with *ompA*-LAMP amplicons simultaneously labeled with FAM and biotin (**Step 2**). Finally, the LAMP amplicons are visually analyzed using LFB within 2 min (**Step 3**).

### The Principle of Visual Detection of *C. trachomatis*-LAMP Products Using LFB

Details of the visual detection of *C. trachomatis*-LAMP products through nanoparticle-based LFB are shown in Figure 2. Notably, 1.0 μl of *C. trachomatis*-LAMP products and 100 μl of running buffer (100 mM PBS, pH 7.4 with 1% Tween 20) were deposited on the sample pad (Figure 2A). The running buffer containing *C. trachomatis*-LAMP products can move along the LFB with capillary action, and rehydrate the immobilized streptavidin-GNPs in conjugate pad (Figure 2B). Positive amplicons were specifically captured by anti-FAM and Biotin-BSA at TL and CL in reaction region, respectively (Figure 2C). For negative results, only streptavidin-DPNs were captured by biotin-BSA at the CL. The interpretation of *C. trachomatis*-LAMP-LFB assay readout is shown in Figure 2D.

### Confirmation and Analysis of *C. trachomatis*-LAMP Products

**Figure 3:**
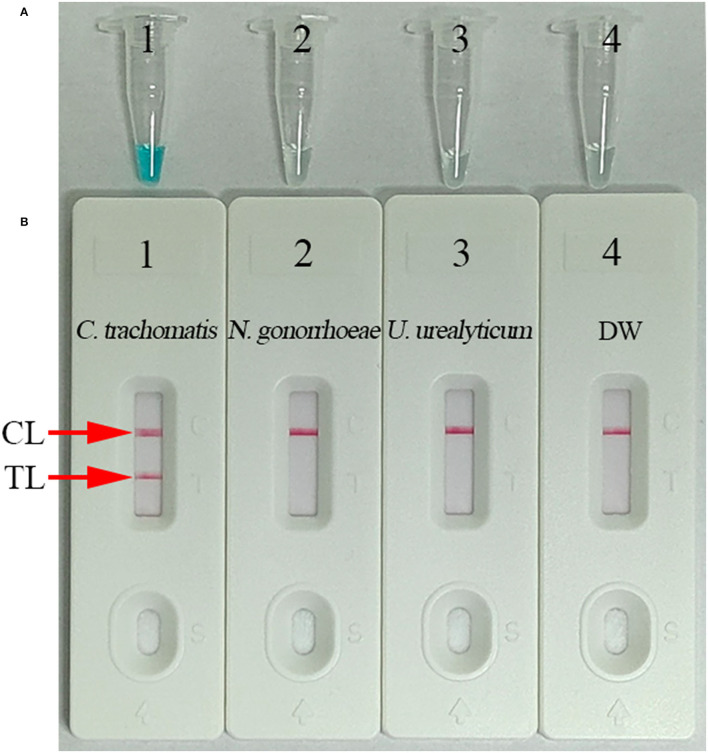
Confirmation and verification of *C. trachomatis*-LAMP products. *C. trachomatis*-LAMP products were measured simultaneously through MG regents **(A)** and LFB **(B)**. Tube 1/biosensor 1: positive result for the *C. trachomatis ompA* standard plasmids; tube 2/biosensor 2: negative result for *Neisseria gonorrhoeae*; tube 3/biosensor 3: negative result for *Ureaplasma urealyticum*; tube 4/biosensor 4: blank control (distilled water, DW). TL, test line; CL, control line.

For validating the *C. trachomatis*-LAMP system, LAMP amplification was performed at a constant temperature of 65°C for 1 h using *ompA* standard plasmids. Using visual MG reagent analysis, the *C. trachomatis*-LAMP tube was visualized by the naked eye as bright green, while the negative controls (*Neisseria gonorrhoeae* and *Ureaplasma urealyticum*) and blank control remained colorless (Figure 3A). In the LFB, two red bands appeared in TL and CL in positive *C. trachomatis*-LAMP amplification. Only a red band presented in CL represents negative and blank controls (Figure 3B). These data demonstrated that the *C. trachomatis*-LAMP primer set and reaction system are valid for target gene detection.

### Confirmation of the Optimal Amplification Temperature for *C. trachomatis*-LAMP-LFB Assay

**Figure 4:**
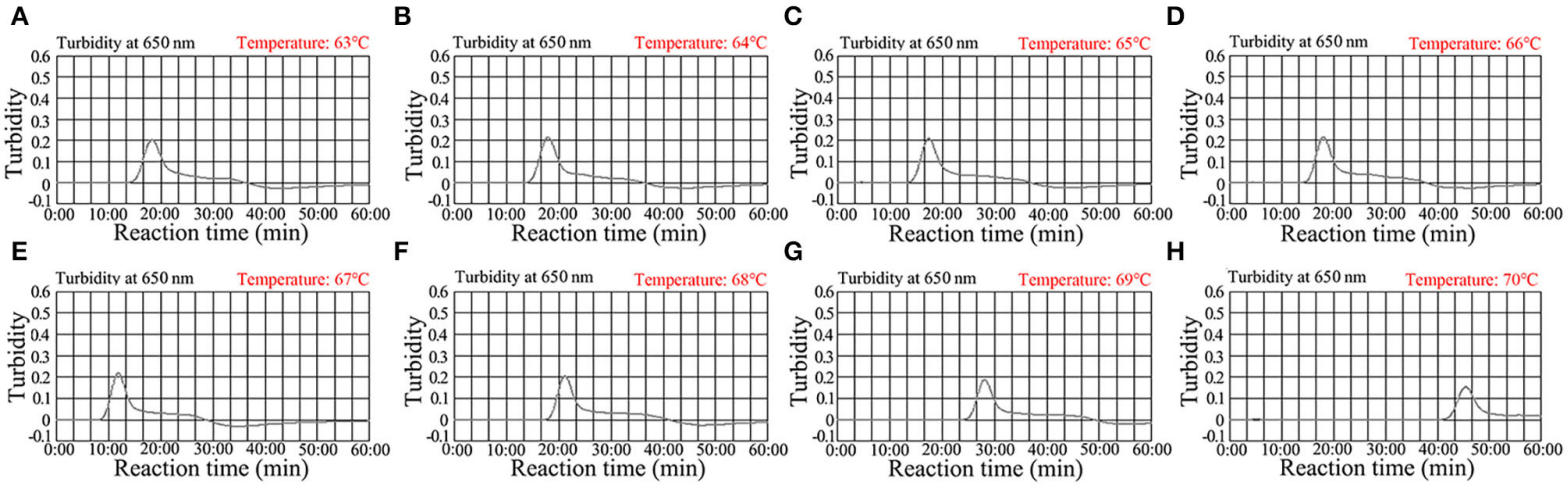
Optimization of the temperature for *C. trachomatis*-LAMP reactions. *C. trachomatis*-LAMP reaction process was monitored using real-time turbidity (LA-500). The threshold value was 0.1, and the turbidity >0.1 was regarded as positive. Eight kinetic graphs **(A–H)** were yielded at various amplification temperatures (63–70°C at 1°C intervals) with *C. trachomatis ompA*-plasmids at the level of 5 × 10^3^ copies/ml. The graphs at 67°C showed robust amplification.

For optimization of the reaction temperature at the *C. trachomatis*-LAMP pre-amplification stage, temperature from 63 to 70°C with 5.0 × 10^3^ copies/ml of *C. trachomatis* DNA template was investigated (Figures 4A–H). Utilizing real-time turbidity (LA-500), the robust amplification of *C. trachomatis*-LAMP was observed at 67°C (Figure 4E). Hence, 67°C was used as the optimal amplification for *C. trachomatis*-LAMP-LFB assay.

### Sensitivity of the *C. trachomatis*-LAMP-LFB Assay

**Figure 5:**
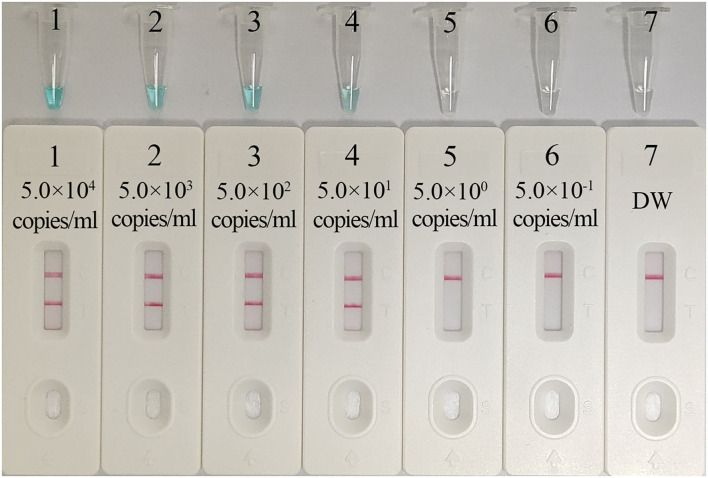
Assay sensitivity using serially diluted *C. trachomatis ompA*-plasmid templates. **(A)** Visual MG regents used for detecting the results; **(B)** LFB used for detecting the results. Tubes A1–A7 (biosensors B1–B7) represent the *C. trachomatis ompA*-plasmid levels of 5.0 × 10^4^, 5.0 × 10^3^, 5.0 × 10^2^, 5.0 × 10^1^, 5.0 × 10^0^, 5.0 × 10^−1^, and 5.0 × 10^−2^ copies/ml and blank control (distilled water). The template levels from 5.0 × 10^4^ to 5.0 × 10^1^ copies/ml showed positive results. CL, control line; TL, test line.

As shown in Figure 5, the *C. trachomatis*-LAMP-LFB assay can detect as few as 50 copies/ml of *ompA* standard plasmids in a vessel. The *C. trachomatis*-LAMP amplification was operated as described above, and the results were obtained using LFB and MG visual reagent. The results obtained *via* LFB were consistent with MG reagent (Figures 5A,B).

### Optimal Amplification Times for the *C. trachomatis*-LAMP-LFB Assay

**Figure 6:**
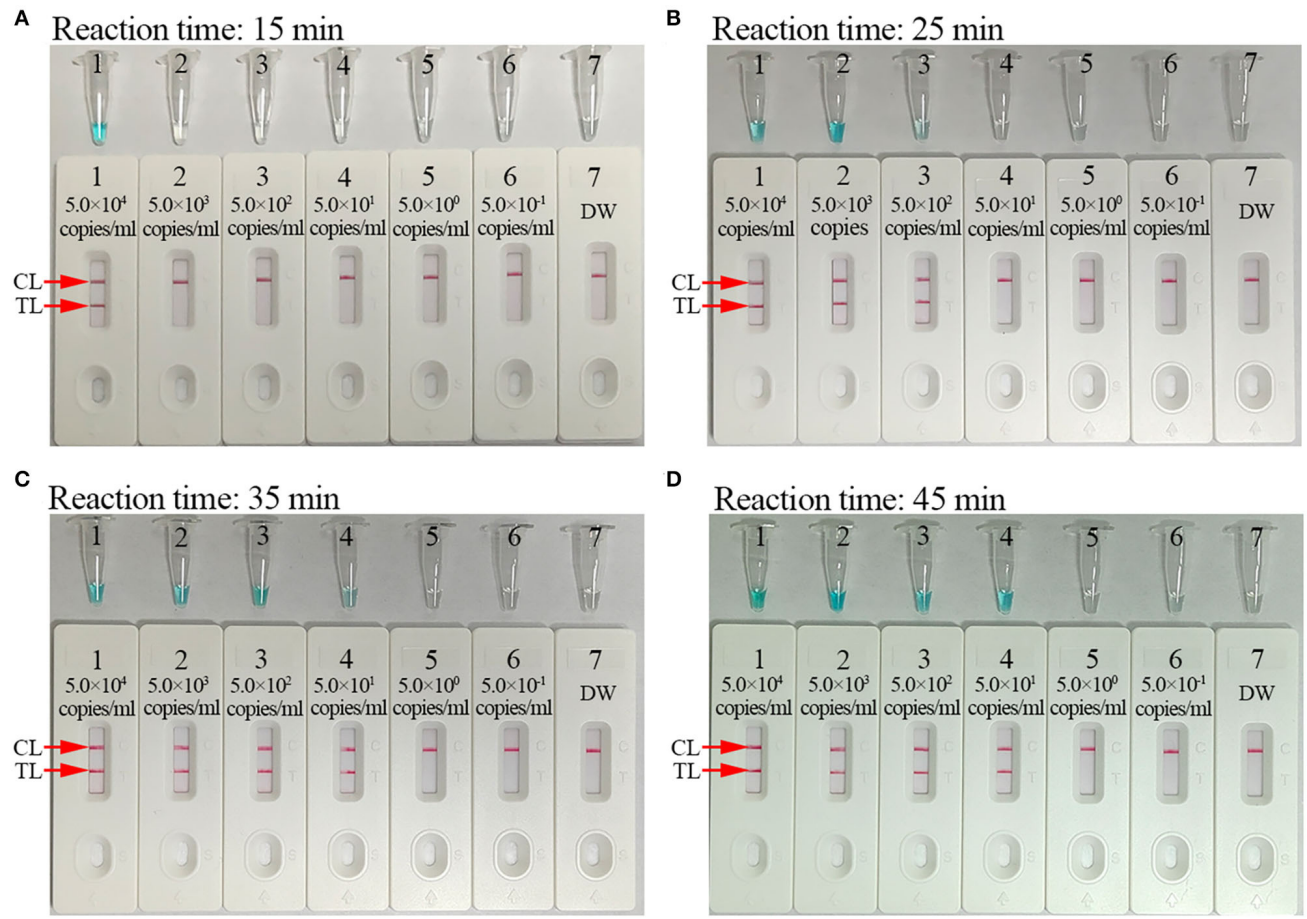
Optimization of reaction time for *C. trachomatis*-LAMP-LFB assay. Reaction times (**A**, 15 min; **B**, 25 min; **C**, 35 min; **D**, 45 min) were tested at optimal reaction temperature of 67°C. Tube/biosensors B1–B7 represent plasmid levels of 5.0 × 10^4^, 5.0 × 10^3^, 5.0 × 10^2^, 5.0 × 10^1^, 5.0 × 10^0^, 5.0 × 10^−1^, and 5.0 × 10^−2^ copies/ml and blank control (distilled water). The LoD of plasmid template could be detected when the reaction lasted for 35 min (C). CL, control line; TL, test line.

The reaction time for the *C. trachomatis*-LAMP-LFB assay during the amplification stage was optimized. As shown in Figure 6, the lowest template level of *ompA* standard plasmid (50 copies/ml) was tested when the reaction time lasted for 35 and 45 min at 67°C, and a reaction time of 35 min was recommended during the LAMP pre-amplification stage. Hence, the whole diagnostic procedure of the *C. trachomatis*-LAMP-LFB technique, including rapid genomic DNA preparation (15 min), LAMP reaction (35 min), and result readout (<2 min), can be completed within 1 h.

### Specificity of the *C. trachomatis*-LAMP-LFB Assay

The specificity estimation of the *C. trachomatis*-LAMP-LFB assay was tested using synthesized templates (*omp A* sequences from serovar A-K, L1, L2, and L3, respectively), *C. trachomatis*-positive clinical samples (confirmed with qPCR), and various non-*C. trachomatis* pathogens (Table 2; Supplementary Figure 2). The positive results were observed when the templates were extracted from *C. trachomatis* species. While no positive results were detected from non-*C. trachomatis* pathogens and blank control (Table 2; Supplementary Figure 2). These data suggested that the *C. trachomatis*-LAMP-LFB assay has an excellent specificity.

### Evaluation of the *C. trachomatis*-LAMP-LFB Assay Using Clinical Samples

A total of 87 suspected *C. trachomatis*-infection genital secretion samples detected were simultaneously using qPCR and *C. trachomatis*-LAMP-LFB assay. A total of 37 (42.5%) were detected as positive outcomes using qPCR (>500 copies/ml); these *C. trachomatis*-positive samples have also been confirmed through *C. trachomatis*-LAMP-LFB. Besides, 3 of the 50 “negative samples” (range from 300 to 500 copies/ml) were detected as positive outcomes through *C. trachomatis*-LAMP-LFB (Table 3; Supplementary Table 1). These results indicated that our *C. trachomatis*-LAMP-LFB assay is an advanced diagnostic tool for suspected *C. trachomatis*-infected patients, especially for those with low bacterial loads.

**Table 3:** Comparison of qPCR and LAMP-LFB for detection of *C. trachomatis* in clinical samples.

**Detection method**	**Clinical samples (*n* = 87)**	
	**Positive**	**Negative**
qPCR	37 (>500 copies/ml)	50 (47, undetected; 3, range from 300 to 500 copies/ml)
LAMP-LFB	40	47

## Discussion

Genital infection with *C. trachomatis* is considered to be one of the most common bacterial STIs worldwide (Ramadhani et al., 2019; La Rosa et al., 2021). A distinguishing feature of *C. trachomatis* infection is that the patients are asymptomatic at the early stage of infection and usually delay or do not seek medical treatment, especially in less-developed regions, which brings about the continued transmission (Tadele et al., 2019; Caven and Carabeo, 2020). Hence, a rapid, accurate, cost-saving, and easy-to-operate assay for early diagnosis of *C. trachomatis* is critical for addressing ever-increasing *C. trachomatis* infection transmission rates. Here, a novel *C. trachomatis*-LAMP-LFB assay, which combined LAMP amplification with gold nanoparticle-based biosensor, was devised and applied successfully to the visual and rapid testing of *C. trachomatis* in clinical samples.

Traditional diagnostic techniques, including cell culture, enzyme immunoassay, and direct fluorescence assay, were used to diagnose *C. trachomatis* infection (Kelly et al., 2017; Peng et al., 2020). However, all of these tools cannot meet fully the requirement of early and easy-to-operate diagnosis in clinical application. Cell culture was regarded as the initial gold standard for *C. trachomatis* detection, but *C. trachomatis* require fastidious and demanding cultivation conditions, and the procedure of cell culture is time-consuming and labor-intensive (Herbst De Cortina et al., 2016). Therefore, it is now seldom utilized in clinical laboratory. Compared with cell cultivation, enzyme immunoassay and direct fluorescence assay are relatively simple and rapid, but are not recommended as routine diagnostic methods due to low diagnostic accuracy (Peng et al., 2020). Nucleic acid amplification tests are more sensitive and have an excellent specificity, which is recommended as the new gold diagnostic method for *C. trachomatis* detection (Herbst De Cortina et al., 2016; Safarkar et al., 2017). However, their use is confined due to requiring complicated laboratory facilities and trained technicians in resource-impoverished regions. In this study, the *C. trachomatis*-LAMP-LFB method only requires simple instruments, such as water bath, heating block, or even a thermos cup that can maintain a constant temperature (67°C). More importantly, the detection results can be visually read out with LFB. The whole diagnosis procedure, including genomic DNA releasing (~15 min), LAMP amplification (35 min), and result visual interpretation (<2 min), can be completed within 60 min.

In this study, LAMP was applied to amplify the target gene of *C. trachomatis*, which is a novel nucleic acid amplification technique devised by Notomi et al., usually 100-fold higher sensitivity than traditional PCR (Notomi et al., 2000; Avendaño and Patarroyo, 2020; Chaouch, 2021). A set of four or six primers spanning six or eight different regions of the target gene makes the amplification results highly specific. The primer set contains two outer primers (F3 and B3), two inner primers (FIP and BIP), and two loop primers (LF and LB) (Panno et al., 2020). Typically, four primers (F3, B3, FIP, and BIP) are enough to amplify a target fragment and improve its efficiency and specificity, while the two loop primers are added to the LAMP amplification system (Augustine et al., 2020; García-Bernalt Diego et al., 2021). In addition, a *Bst* DNA polymerase with chain displacement capability is critical for LAMP amplification at a fixed temperature (Notomi et al., 2000). Here, a set of *C. trachomatis*-LAMP primers were successfully designed for targeting 8 regions of *ompA* gene from 14 *C. trachomatis* serological variants (serovar A, B, C, D, E, F, G, H, I, J, K, L1, L2, L3). The specificity analysis confirmed that the *C. trachomatis*-LAMP-LFB assay can correctly identify the target pathogens and have no cross-reactions with no-*C. trachomatis* templates (Table 2; Supplementary Figure 2). Furthermore, the LoD of *C. trachomatis*-LAMP-LFB assay is as low as 50 copies/ml. To further confirm the feasibility of the assay in clinical application, 87 genomic DNA samples isolated from suspected *C. trachomatis*-infected patients were tested simultaneously with *C. trachomatis*-LAMP-LFB and qPCR. The data indicated that our assay is strengthened in detecting *C. trachomatis*-infected genital secretion samples (Table 3; Supplementar Table 1). To further verify its feasibility, much more low copy number of clinical samples should be collected and tested in the subsequent study. The lower detection rate of qPCR may be attributed to low copy number of the *C. trachomatis* DNA templates, and the qPCR detection was performed with a commercial real-time TaqMan PCR Kit (Da An Gene Co., Ltd. China). The concentrations of *C. trachomatis* < 500 copies/ml will be regarded as a negative outcome according to the illustrations of the manufacturer. In this study, three “negative” clinical specimens (*C. trachomatis* concentrations ranging from 300 to 500 copies/ml) presented positive results using *C. trachomatis*-LAMP-LFB, which further verified that the sensitivity of our assay is higher than the traditional PCR technique.

For visual and rapid presentation of *C. trachomatis*-LAMP results, gold nanoparticle-based LFB was used in this assay system. LFB is a paper-based device that has been extensively used in the diagnosis of infectious diseases, foodborne pathogens, cancer biomarkers, and cardiovascular disease, owing to its low-cost, rapid detection with good robustness, specificity, sensitivity, and easy-to-operate (Hsieh et al., 2017; Huang et al., 2020; Wang et al., 2021). Here, the LFB strips were immobilized with anti-FAM and BSA-biotin on TL and CL, respectively. For positive outcomes, the FAM/biotin-labeled *C. trachomatis*-LAMP amplicons were arrested by anti-FAM at the TL and the streptavidin-DPNs were captured by biotin-BSA at the CL, respectively. For negative results, only the streptavidin-DPNs were arrested by biotin-BSA at the CL. Although the MG regents and real-time turbidity were able to test the *C. trachomatis*-LAMP results in this study, the former is ambiguous when LAMP amplicon concentrations were low (Figure 6C), and the latter technique requires expensive apparatus. The LFB method is simple and cost-effective (~US$2.0 for each strip). Hence, the overall cost of each *C. trachomatis*-LAMP-LFB test, including DNA template extraction (~US$1.0), LAMP reaction (~US$3.0), and LFB readout (~US$2.0), was calculated at US$6.0. In previous studies, LAMP has already been used to identify *C. trachomatis*. Choopara et al. (2017) and Somboonna and Choopara (2019) combined LAMP with hydroxynaphthol blue for visual color detection of *C. trachomatis* by the naked eye, but the results of this assay were ambiguous when LAMP product concentrations were low. Dean et al. (2021) have also utilized the LAMP assay for testing *C. trachomatis*, and the *C. trachomatis*-LAMP amplification products were examined with colorimetric chemistry, which must rely on optical detection instrumentation. In this study, we first combined LAMP amplification with LFB for the identification of *C. trachomatis*, which is more convenient and rapid than other methods.

The *C. trachomatis*-LAMP-LFB also has some shortcomings. First, there is a risk of carryover contamination; the LAMP reaction tube must be taken off for LFB identification. Spraying a 10–15% sodium hypochlorite solution and 70% ethanol after LFB detection is an effective way of avoiding nucleic acid contamination in laboratory. In this study, no false-positive outcomes were observed in non-*C. trachomatis* templates. It is indicated that the cross-contamination can be effectively controlled in our laboratory. Secondly, our assay can be used for qualitative detection of *C. trachomatis*, but not measurement of the concentrations of *C. trachomatis* in sample; the quantitative determination of LAMP-LFB could be considered for further study in the future. Thirdly, the *C. trachomatis*-LAMP-LFB system just detected the *ompA* gene from 14 *C. trachomatis* serological variants (serovar A-K, L1, L2, L3) and neglected the internal control gene. For better fitting the clinical application, the *C. trachomatis*-LAMP amplification system should add a set of LAMP primers based on the internal control gene (human gene) for avoiding false-negative results. The LFB has designed three lines, including CL, internal control line, and TL, which can effectively avoid false-positive results.

In conclusion, we combined a LAMP reaction with LFB readout platform for successfully devising a novel *C. trachomatis*-LAMP-LFB for the rapid, visual, cost-effective, specific, and sensitive identification of *C. trachomatis* agent in clinical samples. The LoD of our assay was 50 copies/ml and had no cross-reaction with non-*C. trachomatis* pathogens. The whole assay process can be completed within 60 min without any special instrument. Therefore, the *C. trachomatis*-LAMP-LFB shows great potential as POC testing for *C. trachomatis* screening and detection in clinical settings, especially in resource-starved regions.

## Data Availability Statement

The datasets presented in this study can be found in online repositories. The names of the repository/repositories and accession number(s) can be found in the article/Supplementary Material.

## Ethics Statement

The study was approved by the Human Ethics Committee of Hangzhou Women's Hospital (Approval No. [2021]-K (2)-8) and complied with the Declaration of Helsinki. Before clinical samples/isolates were obtained, all personal patient identifiers were removed. Patients' informed consent was waived by the committee.

## Author Contributions

XC and SD designed and conceived this study and drafted and revised the manuscript. XC, QZ, YT, RW, XW, JL, RL, SW, and SD collected clinical samples and performed the experiments. XC, QZ, and SD analyzed the data. All authors read and approved the final manuscript.

## Funding

This study was funded by the Program of Scientific and Technological Project in Guizhou Province (Grant No. Qian Ke He [2020]4Y184), the Scientific and Technological in Guiyang City (Grant No. Zhu Ke He [2020]-10-5), and the Public Welfare Technology Research Program in Zhejiang Province (Grant No. LGF21H190001).

## Conflict of Interest

The authors declare that the research was conducted in the absence of any commercial or financial relationships that could be construed as a potential conflict of interest.

## Publisher's Note

All claims expressed in this article are solely those of the authors and do not necessarily represent those of their affiliated organizations, or those of the publisher, the editors and the reviewers. Any product that may be evaluated in this article, or claim that may be made by its manufacturer, is not guaranteed or endorsed by the publisher.

## Abbreviations

STIs: sexually transmitted infections
LAMP: loop-mediated isothermal amplification
LFB: gold nanoparticle-based lateral flow biosensor
WHO: World Health Organization
PCR: polymerase chain reaction
MG: malachite green
LoD: limit of detection
2nd GZUTCM: Second Affiliated Hospital, Guizhou University of Traditional Chinese Medicine
GZCCL: Guizhou Provincial Center for Clinical Laboratory
POC: Point of care
nt: nucleotide
mer: monomeric unit
FAM: carboxyfluorescein
TL: test line
CL: control line
DW: distilled water
BC: blank control.
