# Supplementary material for: Nanoparticle-Based Lateral Flow Biosensor Integrated With Loop-Mediated Isothermal Amplification for Rapid and Visual Identification of Chlamydia trachomatis for Point-of-Care Use
Source: Front Microbiol. 2022 Jul 12;13:914620. doi: 10.3389/fmicb.2022.914620 (PMC9318599; doi:10.3389/fmicb.2022.914620)
Supplement: Supplementary file 1 [file Table_1.docx]

**Supplementary Materials**

**Nanoparticle-based lateral flow biosensor integrated with loop-mediated isothermal amplification for rapid and visual identification of *Chlamydia trachomatis* for point-of-care use**

Xu Chen^1,2^, Qingxue Zhou^3^, Yan Tan^4^, Ronghua Wang^5^, Xueli Wu^2^, Jiangli Liu^2^, Rui Liu^2^, Shuoshi Wang^2^, Shilei Dong^6*^

1. The Second Clinical College, Guizhou University of Traditional Chinese Medicine, Guiyang, Guizhou, 550003, People’s Republic of China
2. Clinical Medical Laboratory of the Second Affiliated Hospital, Guizhou University of Traditional Chinese Medicine, Guiyang, Guizhou, 550003, People’s Republic of China
3. Clinical Laboratory, Hangzhou Women’s Hospital, Hangzhou, Zhejiang 310008, People’s Republic of China
4. Guizhou Provincial Center for Clinical Laboratory, Guiyang, Guizhou, 550002, People’s Republic of China
5. Department of Clinical Laboratory, Longli people’s Hospital, Qianlan, Guizhou, 551299, People’s Republic of China
6. Department of Clinical Laboratory, Zhejiang Hospital, Hangzhou, Zhejiang, 310013, People’s Republic of China

*Corresponding author:

Shilei Dong, E-mail: dsl166@126.com

**FIGURE S1 Nucleotide sequences and location of the *ompA* gene used to design the *C. trachomatis-*LAMP primers**

The nucleotide sequences of the *ompA* gene from 14 *C. trachomatis* serological variants (serovar A, B, C, D, E, F, G, H, I, J, K, L1, L2, L3) were aligned with MEGA-X software, and the LAMP primer sequences are marked with arrows. The right and left arrows showed sense and complementary sequences, respectively.

**FIGURE S2 Assay specificity using different pathogens**

The specificity of *C. trachomatis*-LAMP-LFB assay was tested using various pathogens. Biosensor 1-14, *C. trachomatis* serovar A, B, C, D, E, F, G, H, I, J, K, L1, L2, and L3 *ompA*-plasmids; Biosensor 15-19, *C. trachomatis* (clinical samples); Biosensor 20, *Neisseria gonorrhoeae*; Biosensor 21, *Ureaplasma urealyticum*; Biosensor 22, *Mycobacterium tuberculosis*; Biosensor 23, *Escherichia coli*; Biosensor 24, *Haemophilus influenza*; Biosensor 25, *Cryptococcus neoformans*; Biosensor 26, *Streptococcus pyogenes*; Biosensor 27, *Pseudomonas aeruginosa*; Biosensor 28, *Staphylococcus aureus*; Biosensor 29, *Candida glabrata*; Biosensor 30, *Bordetella pertussis*; Biosensor 31, *Hemophililus parainfluenza*; Biosensor 32, *Klebsiella pneumonia*; Biosensor 33, *Mycoplasma pneumonia*; Biosensor 34, *Shigella flexneri*; Biosensor 35, *Listeria monocytogenes*; Biosensor 36, Human enterovirus EV71; Biosensor 37, Coxsackie virus CAV16; Biosensor 38, Human rhinovirus; Biosensor 39, Human papilloma virus; Biosensor 40, blank control (distilled water). CL: control line; TL: test line.

**TABLE S1:** Comparison of qPCR, and LAMP-LFB assays for diagnosis of *C. trachomatis* using clinical samples

| **Sample NO.** | **qPCR results（copies/ml）** | **LAMP-LFB results** |
| --- | --- | --- |
| Test 1 | 4.98×10^5^ | + |
| Test 2 | 655 | + |
| Test 3 | 9.32×10^5^ | + |
| Test 4 | 1.53×10^3^ | + |
| Test 5 | 5.86×10^4^ | + |
| Test 6 | 5.06×10^3^ | + |
| **Test 7** | **— (~340)** | **+** |
| Test 8 | 2.82×10^6^ | + |
| Test 9 | 4.44×10^5^ | + |
| Test 10 | 1.69×10^4^ | + |
| Test 11 | 1.74×10^4^ | + |
| Test 12 | 2.30×10^4^ | + |
| Test 13 | 4.75×10^3^ | + |
| Test 14 | 552 | + |
| Test 15 | 1.87×10^3^ | + |
| Test 16 | 1.04×10^3^ | + |
| Test 17 | 5.82×10^3^ | + |
| Test 18 | 1.89×10^3^ | + |
| Test 19 | 646 | + |
| Test 20 | 2.45×10^3^ | + |
| Test 21 | 1.14×10^5^ | + |
| Test 22 | 9.36×10^4^ | + |
| **Test 23**  Test 24  Test 25  Test 26  Test 27  Test 28  **Test 29**  Test 30  Test 31  Test 32  Test 33  Test 34  Test 35  Test 36  Test 37  Test 38  Test 39  Test 40 | **— (~300)**  1.89×10^5^  1.62×10^5^  2.45×10^3^  1.14×10^3^  9.36×10^3^  **— (~320)**  3.62×10^4^  8.85×10^4^  1.39×10^5^  3.47×10^4^  4.92×10^6^  728  1.21×10^5^  1.66×10^3^  1.34×10^4^  7.13×10^5^  1.24×10^6^ | **+**  +  +  +  +  +  **+**  +  +  +  +  +  +  +  +  +  +  + |
| Test 41-87 | — | — |

**Notice:** The qPCR diagnosis was carried out using commercial real-time TaqMan PCR Kit (DaAn Gene Co., Ltd. China). The concentrations of *C. trachomatis* less than 500 copies/ml will be regarded as negative outcome according to the manufacturer’s instructions.

+, Positive; —, Negative
