# Supplementary figures and images for: Nanoparticle-Based Lateral Flow Biosensor Integrated With Loop-Mediated Isothermal Amplification for Rapid and Visual Identification of Chlamydia trachomatis for Point-of-Care Use
Source: Front Microbiol. 2022 Jul 12;13:914620. doi: 10.3389/fmicb.2022.914620 (PMC9318599; doi:10.3389/fmicb.2022.914620)

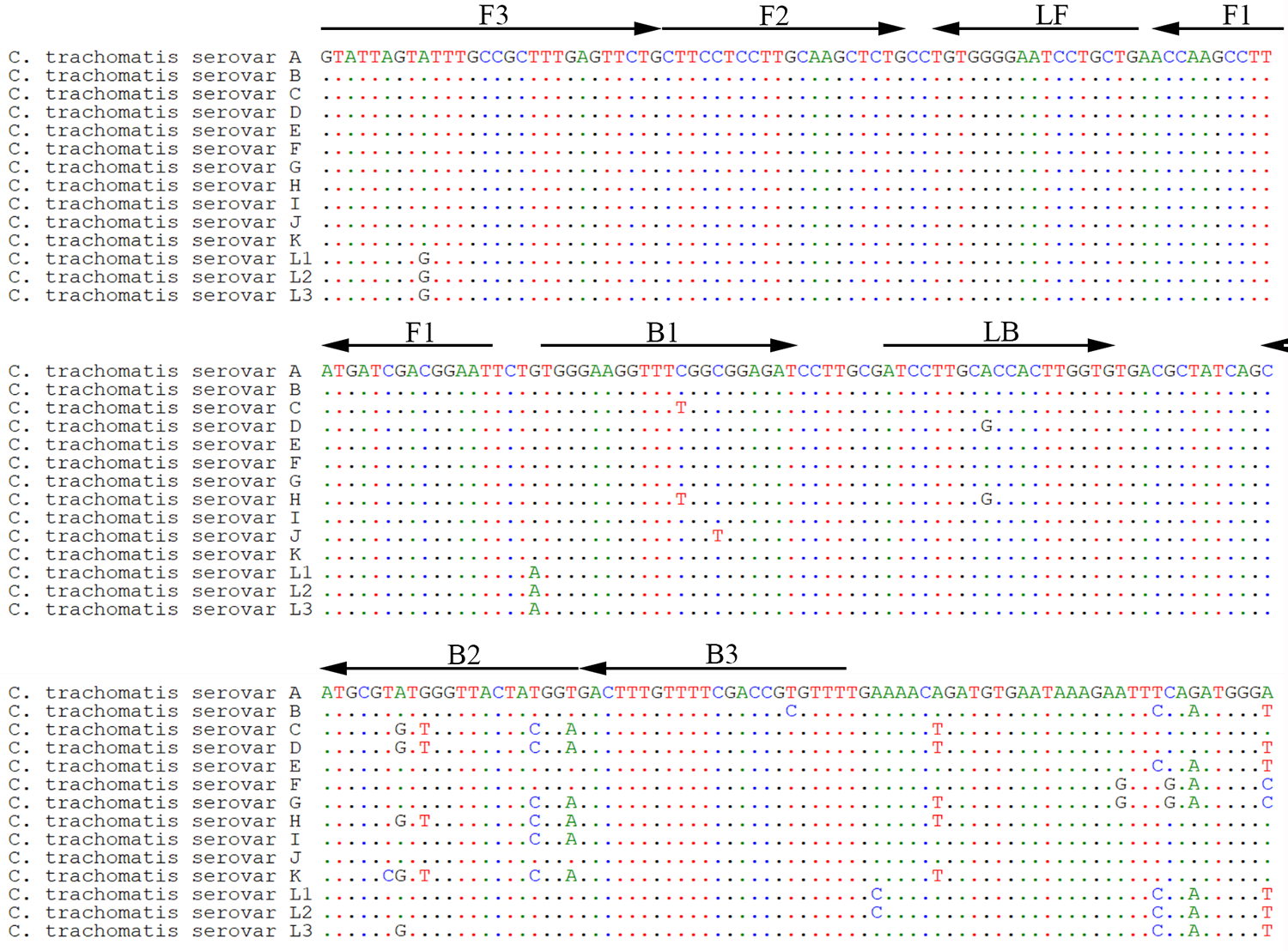

Supplement: Supplementary file 2 [file Image_1.TIF]

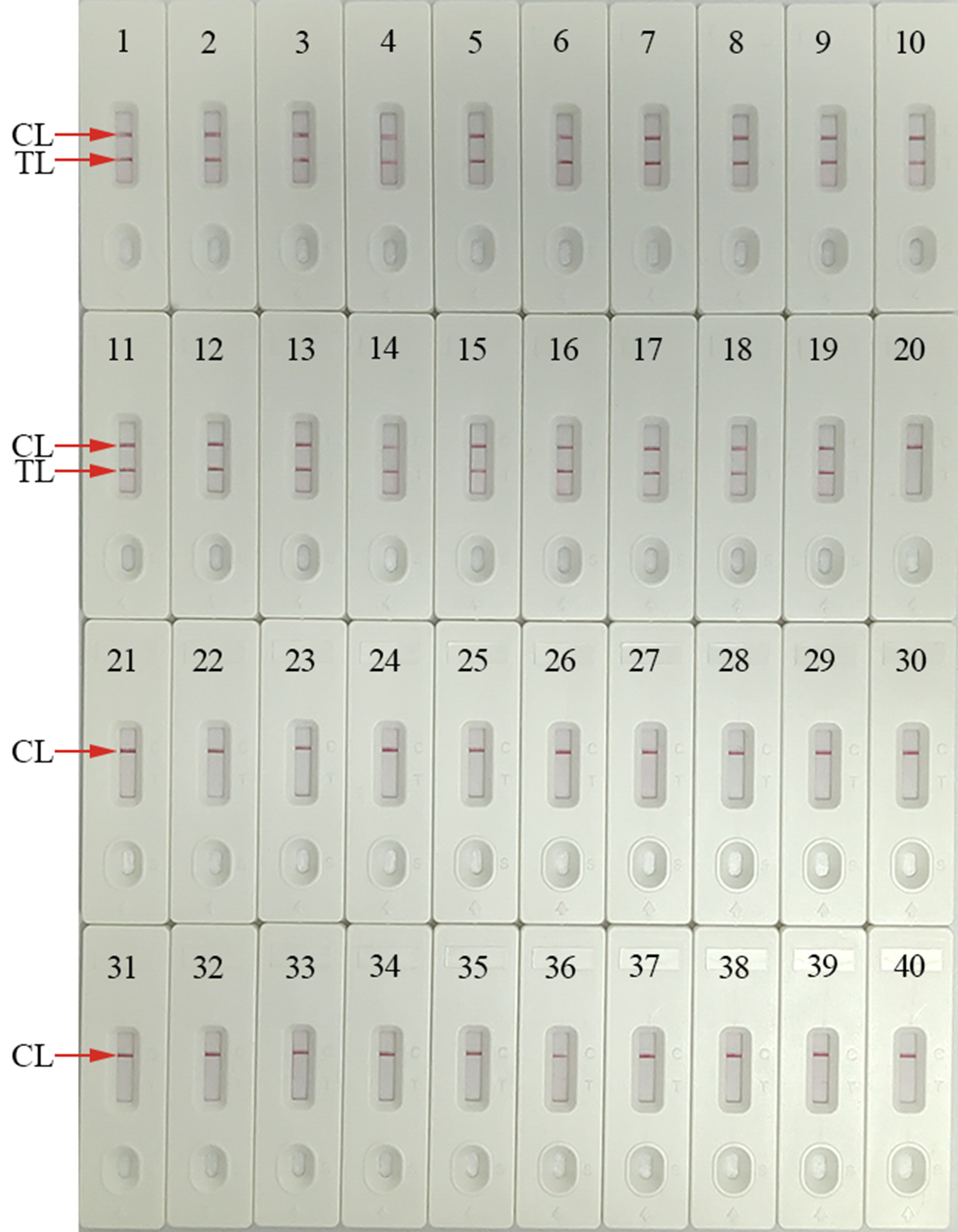

Supplement: Supplementary file 3 [file Image_2.TIF]
